# Multi-Excitation Infrared Fusion for Impact Evaluation of Aluminium-BFRP/GFRP Hybrid Composites

**DOI:** 10.3390/s21175961

**Published:** 2021-09-05

**Authors:** Jue Hu, Hai Zhang, Stefano Sfarra, Stefano Perilli, Claudia Sergi, Fabrizio Sarasini, Xavier Maldague

**Affiliations:** 1 School of Automation Engineering, University of Electronic Science and Technology of China, Chengdu 611731, China; jue.hu.2@ulaval.ca; 2 Department of Electrical and Computer Engineering, Computer Vision and Systems Laboratory, Laval University, Quebec, QC G1V 0A6, Canada; hai.zhang.1@ulaval.ca (H.Z.); xavier.maldague@gel.ulaval.ca (X.M.); 3 Department of Industrial and Information Engineering and Economics (DIIIE), University of L’Aquila, Monteluco di Roio, 67100 L’Aquila, Italy; 4 Independent Researcher, 67100 Santa Rufina di Roio, Italy; stefanoperilli.ps@gmail.com; 5 Department of Chemical Engineering Materials Environment, Sapienza-Università di Roma & UdR INSTM, 00184 Roma, Italy; claudia.sergi@uniroma1.it (C.S.); fabrizio.sarasini@uniroma1.it (F.S.)

**Keywords:** non-destructive testing, infrared thermography, fibre metal laminates, feature fusion

## Abstract

Fibre metal laminates are widely implemented in the aerospace industry owing to the merits of fatigue resistance and plastic properties. An effective defect assessment technique needs to be investigated for this type of composite materials. In order to achieve accurate impact-induced damage evaluation, a multi-excitation infrared fusion method is introduced in this study. Optical excitation thermography with high performance on revealing surface and subsurface defects is combined with vibro-thermography to improve the capability of detection on defects. Quantitative analysis is carried out on the temperature curve to assess the impact-induced deformation. A new image fusion framework including feature extraction, feature selection and fusion steps is proposed to fully utilize the information from two excitation modalities. Six fibre metal laminates which contain aluminium-basalt fibre reinforced plastic and aluminium-glass fibre reinforced plastic are investigated. Features from different perspectives are compared and selected via intensity contrast on deformation area for fusion imaging. Both types of defects (i.e., surface and sub-surface) and the internal deformation situation of these six samples are characterized clearly and intuitively.

## 1. Introduction

Fibre metal laminate (FML) is a type of composite structure that is fabricated with stacked layers of fabric epoxy prepreg and metal sheets [1,2]. This structure combines the fatigue resistance of fibre-reinforced materials with the plastic property of metal sheet layers. Owing to these merits, FMLs are widely implemented in the aerospace industry as the candidates to replace traditional alloys. Besides, concrete-filled glass fibre reinforced polymer-steel composite tube columns were found to have better maximum bearing capacity than concrete-filled steel tube columns in a recent study [3]. This proves that fibre metal composite structures have a great implementation potential in construction and building materials. Serving as the alternatives in the key equipment requires powerful non-destructive testing methods to ensure safety. To achieve this goal, several non-destructive testing techniques have been investigated on FMLs in recent years [4]. Farias et al. used guided Lamb wave for inspection on the fibre crack and delamination existing on the aluminium laminated and carbon fibre reinforced composites [5]. Time and frequency analysis were both carried out on the guided wave signals and the k-means clustering method was used as the classifier for decision making on the frequency components. Soltannia et al. adapted vibration testing on the nanocarbon particles-reinforced FMLs to investigate the characteristics of two different structures [6]. Here, Fast Fourier transform (FFT) was used to acquire the signal spectrum and the peak amplitude was selected as the feature to characterize the structure properties. Tang et al. introduced the vision-based deformation and strain detection method for concrete-filled steel tube columns [7]. Four ocular stereoscopic vision systems were designed to conduct full-field surface deformation measurement. This work was further developed with an adaptive point-cloud correlation algorithm [8] to further reduce the global calibration error. High accuracy 3D reconstruction was achieved on the multi-vision system.

Infrared thermography (IRT) as a fast, contactless, and high-resolution imaging technique has attracted a wide range of researchers to investigate its application as an efficient non-destructive testing (NDT) technique. IRT involves different thermal excitation methods, e.g., optical excitation thermography (OET), laser excitation thermography, vibro-thermography (VT), and induction thermography [9,10]. In OET, the surface of the sample is heated by a flash lamp array or halogen lamps. Meanwhile, the thermal sequence of the subsequent cooling stage of the surface is recorded by a thermal camera. OET is a non-contact technique with a relatively large inspecting area [11]. Montanini et al. used OET in lock-in mode for inspection of the inserts with different geometries and depths in thick glass fibre reinforced plastic plates [12]. Zhang et al. implemented OET for image diagnosis on dry carbon fibre preforms of three carbon fibre fabrics [13]. The carbon fibre fabrics were made of twill weave, plain weave, and unidirectional stitch. Four image processing algorithms were compared using capability of detection (CoD) analysis to quantify their capability. Laser excitation thermography uses a laser beam as a thermal source. This excitation method is more suitable for the micro-sized flaws detection task in composites, considering the structural complexity which leads to abnormal thermal diffusion when conventional IRT techniques are adapted [14]. Montinaro et al. used flying laser spot thermography to detect the disbonds in FML. The sample was made of aluminium and glass fibre reinforced plastic [15]. A laser beam from a continuous wave laser source was selected for heating and the experiment was carried out using the linear scan mode. Standard derivation of temperature on the reference area was analyzed as the main feature to locate the disbonds on the sample. In VT, the sample is excited by an elastic wave from mechanical vibration. The mechanical energy is converted into thermal energy during the propagation of the damp acoustic waves, while the energy dissipation in the vicinity of defects is more obvious due to the friction at the surrounding area [16,17,18]. Induction thermography is a non-contact, real-time measurement method over a large region, which is effective for the evaluation on the composites with electrical conductivity, such as carbon fiber reinforced plastic (CFRP) [19,20].

As illustrated above, each kind of excitation method of IRT has its own properties and suitable situation for implementation. Inspired by the study described in [21], Montinaro et al. adapted three types of IRT methods for evaluation of the fluorinated ethylene propylene (FEP) film inserts on the titanium-graphite FML. However, these three types of IRT methods were conducted separately. A more integrated assessment can be developed. Besides, damages caused by actual in-service process need to be investigated. In this study, six FMLs, containing aluminium-basalt fibre reinforced plastic (BFRP) and aluminium-glass fibre reinforced plastic (GFRP), are investigated for the impact-induced damage evaluation. OET with a high performance on revealing surface and subsurface defects is combined with VT to improve the capability of defect detection and achieve accurate evaluation on the materials. Quantitative analysis is carried out on the temperature curve to assess the impact-induced deformation. In order to fully utilize the information of two excitation modalities, we are proposing a new image fusion framework which contains feature extraction, feature selection and fusion steps to extract effective defect information and merge the information for accurate diagnosis. Feature extraction techniques are carried out to obtain the features from time, frequency, and statistical perspectives. Feature selection based on the intensity contrast of the deformation area is implemented to select the appropriate feature image for the fusion step. Feature images selected from two excitation modalities are fused to generate the fusion results, which could characterize both types of defects (i.e., surface and sub-surface) and the internal deformation situation intuitively.

## 2. Specimens

**Figure 1:**
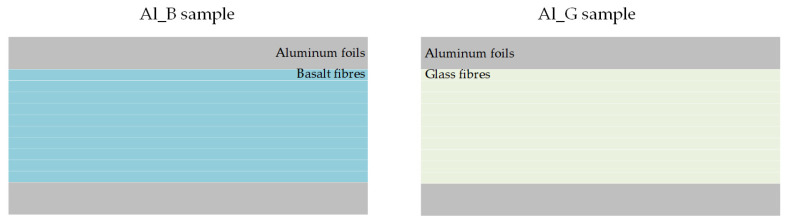
Schematic structures of the specimens.

In order to investigate the damage caused by real in-service process, fibre metal laminates need to be processed with actual impact experiments. As shown in Figure 1, the fibre metal laminates produced for this research were designed with a stacking sequence of aluminium foils and a group composed of composite layers [22].

The aluminium foils in the FMLs are 2024-T3 foils with a thickness of 0.6 mm. The group of composite layers refers to the BFRP/GFRP, which contains 10 layers of basalt (areal density = 220 $g/m^{2}$) /glass (areal density = 204 $g/m^{2}$) plain woven fabrics in a polypropylene (PP) matrix (Hyosung Topilene PP J640, Seoul, Republic of Korea). The mechanical properties of raw materials for the FMLs are summarized in Table 1.

The PP was modified with two PP-g-MA coupling agents from Chemtura in two amounts (wt%), namely Polybond 3200 (2 wt%) and Polybond 3000 (5 wt%). PP modified with Polybond 3000 was used in contact with the aluminium layers, while PP modified with Polybond 3200 was used to impregnate basalt and glass fibres. The stack was then heated to 210 °C under a pressure of 4 MPa in a Collin GmbH (Maitenbeth, Germany) mod. P400E press. The cooling time was 10 min. The actual content of the fibres and the matrix, as well as the void content, were evaluated according to the ASTM D 3171 standard on each tested laminate as shown in Table 2.

**Figure 2:**
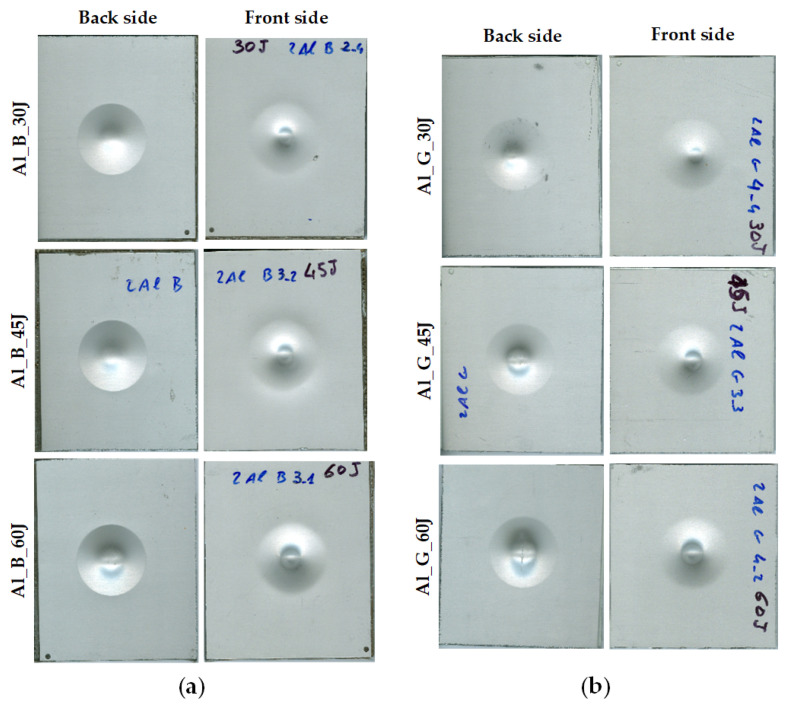
Photographs of the six samples: (**a**) Aluminium-BFRP hybrid composites; (**b**) Aluminium-GFRP composites.

A drop weight impact tower (Ceast/Instron 9350, Instron, Italy) was used to induce damage in FMLs. The samples were clamped between two steel plates with a circular central opening in the impact fixture of diameter equal to 40 mm. A hemispherical striker with a diameter of 20 mm and a total mass of 13 kg were used to deliver impact energy levels of 30 J, 45 J, and 60 J. Figure 2 illustrates the photography of six specimens. Figure 2 indicates that, with the increase of impact energy level, the central area of the deformation changed obviously, and a crack appeared on the aluminium layer on the back side. For characterization of the deformation and internal defects, infrared thermography experiments were carried out with multiple excitation approaches to acquire defect information.

## 3. Methodology

### 3.1. Experimental Setup

In order to achieve accurate impact-induced damage evaluation, multi-excitation thermography is carried out to collect complementary defect information. As is well-known, OET has a high performance with respect to the revealing surface and subsurface defects considering its characteristic of uniform heating. Besides, the VT has the capability of obtaining the internal structure of the sample due to the heat flow transferring from the inside to the surface. Therefore, it could be reasonable to select OET and VT to provide surface and internal information as excitation modalities for multi-excitation thermography of the FMLs.

As an OET approach for IRT, pulsed thermography (PT) uses a flash lamps array with high-energy to generate uniform heating on the surface of specimens. After excitation, the heat transmits through the specimen by diffusion. As time elapses, the surface temperature decreases uniformly for a specimen without internal flaws. On the contrary, surface and sub-surface discontinuities act as resistances to heat flow, which change the diffusion rate and produce abnormal temperature patterns. As a time-domain sequenced imaging approach, PT allows the implementation of advanced imaging processing techniques to extract more visible imprints of defects.

**Figure 3:**
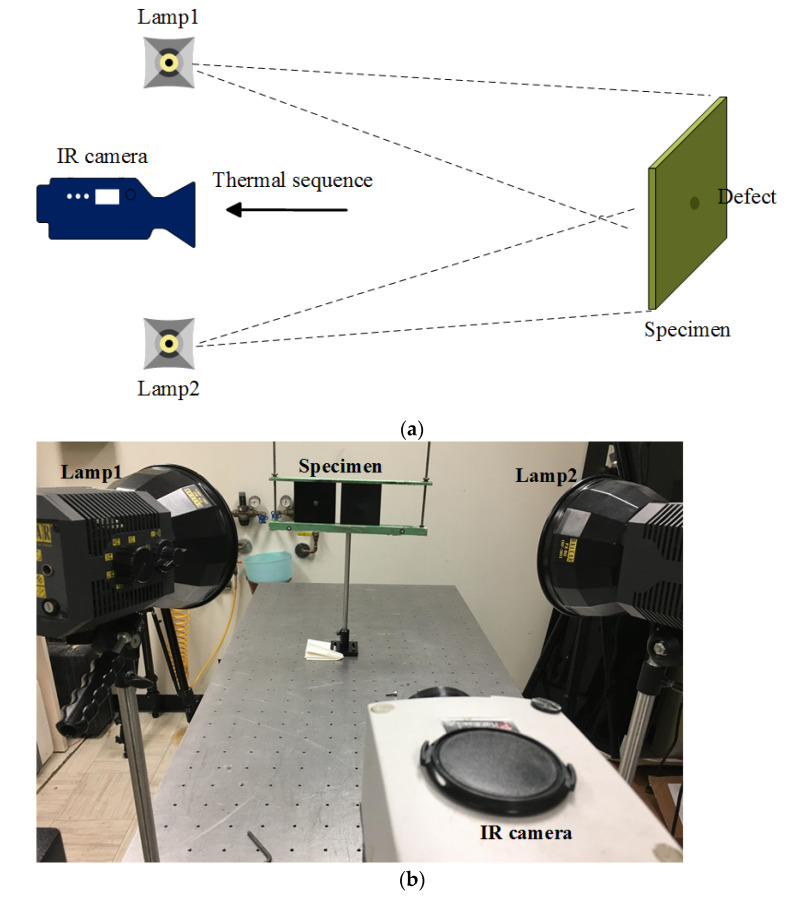
Optical excitation thermography inspection: (**a**) schematic configuration; (**b**) experimental set-up.

Figure 3 shows the schematic configuration and experimental set-up for PT. Two flash units (Balcar FX60, 6.4 kJ, 2 ms duration) were used to generate optical flashes in this configuration. A mid-wave IR camera (FLIR Phoenix) with a frame rate of ~55 Hz and the NETD of 25 mK was adapted to record the temperature profile. The cooling time was set at 10 s for flashes. The camera spatial resolution is 640 × 512 pixels (25 μm × 25 μm of detector size) and a 50 mm lens was employed to provide a field of view (FOV) of 18.2° (horizontal) × 14.6° (vertical).

**Figure 4:**
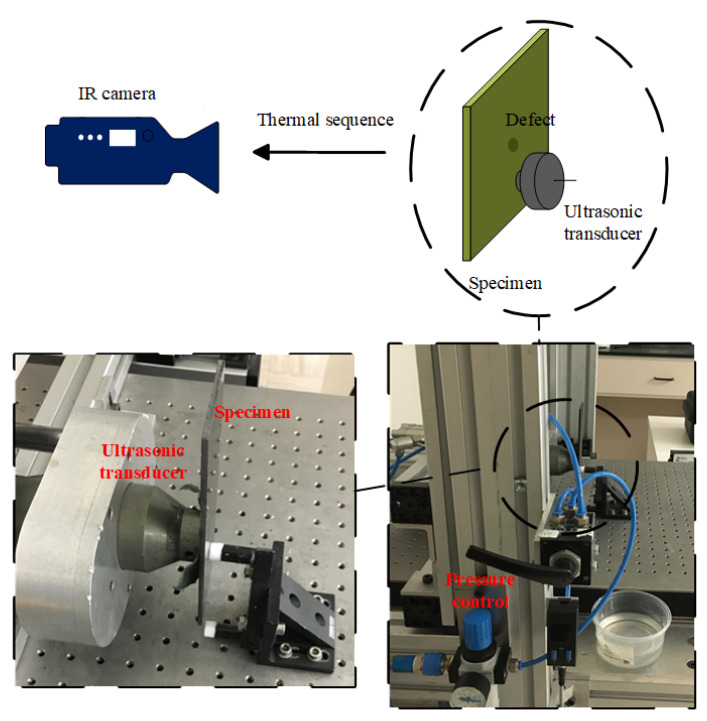
Experimental configuration for vibro-thermography inspection.

Vibro-thermography was also conducted as shown in Figure 4. Both the same IR camera and frame rate (55 Hz) as in PT was used in the vibro-thermography experiment. The transducer was pressed against each specimen, and two periods of 0.2 Hz (10 s) lock-in ultrasonic waves were delivered. Owing to the excitation method, vibro-thermography has a stronger capability with respect to internal structure inspection than PT. Correspondingly, this type of IRT approach sacrifices the surface and sub-surface information in comparison with PT.

### 3.2. FE-S-F Framework

**Figure 5:**
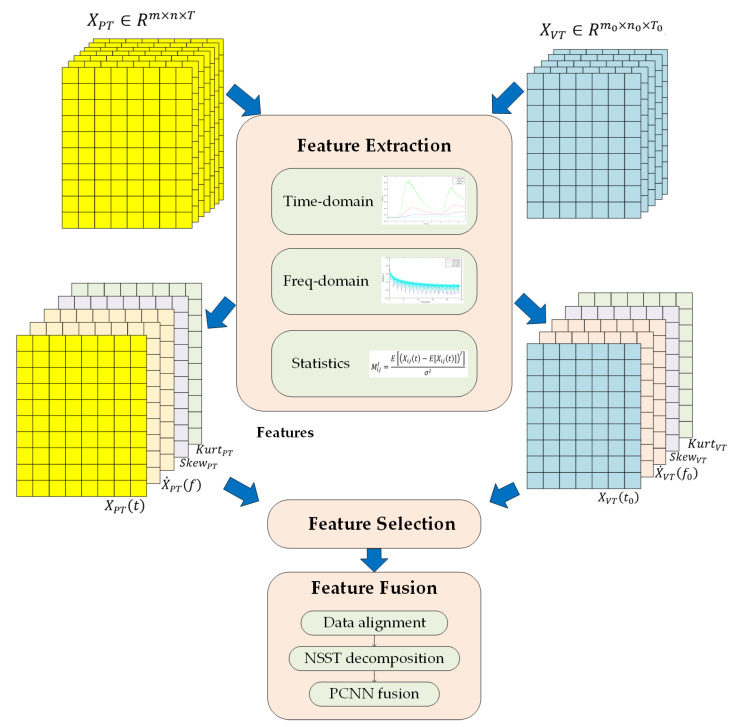
The schematic diagram of the proposed FE-S-F framework for multi-excitation infrared fusion.

In order to fully utilize the information of two excitation modalities, a framework containing feature extraction, feature selection, and fusion (FE-S-F) is proposed for the defect information extraction and merging. The diagram of the proposed FE-S-F framework is shown in Figure 5, and all the processing was conducted by Matlab.

First of all, the raw data collected from IRT experiments using two types of excitations are processed with feature extraction methods to obtain the features from time, frequency, and statistical perspectives. Meanwhile, quantitative analysis is carried out on the temperature curve on the typical frame to obtain information regarding the deformation area. Then, feature selection based on the intensity contrast of deformation area is implemented to select the appropriate feature image for the fusion step. Finally, the features from two excitation modalities are fused in non-subsampled shearlet transform (NSST) domain with pulse-coupled neural network (PCNN) to generate the fusion image with defect information of the inspection using two excitation modalities [23].

#### 3.2.1. Feature Extraction Stage

In the feature extraction stage, the data collected from the PT system is denoted as $X_{PT}\in R^{m\times n\times T}$, where m and n refer to the number of pixels on row and columns of the thermal images. T represents the number of thermal frames in the sequence recorded by the thermal camera. Data from the VT system is represented as $X_{VT}\in R^{m_{0}\times n_{0\;}\times T_{0}}$. Similarly to the marks on PT data, $m_{0}$, $n_{0}$, and $T_{0}$ refer to the size of input thermal sequence, respectively. The time domain features $X_{PT}\left(t\right)\in R^{m\times n}$ and $X_{VT}\left(t_{0}\right)\in R^{m_{0\;}\times n_{0}}$ are the typical frames extracted from the thermal sequence, especially the several frames after the excitation process. Here, t=0,…,T and $t_{0}=0,\ldots ,T_{0}$ refer to the corresponding time points of typical frames. The frequency domain features are obtained through the Fourier analysis on the temperature variation waveform of a single pixel:

(1) $${\dot{X}}_{ij}\left(f\right)=\sum_{0}^{T}X_{ij}\left(t\right)e^{-\frac{i2\pi ft}{T}},$$

where $X_{ij}\left(t\right)$ refers to the temperature variation waveform at point (i,j) of $X_{PT}$. Here, i=1,…,m and j=1,…,n refer to the location of the point. ${\dot{X}}_{ij}\left(f\right)$ denotes the corresponding frequency domain representation of this point. After the calculation of the frequency domain representation point-by-point, the frequency domain features ${\dot{X}}_{PT}\left(f\right)\in R^{m\times n}$ could be reconstructed. The same procedure is also conducted on the VT data to obtain the frequency domain features ${\dot{X}}_{VT}\left(f_{0}\right)\in R^{m_{0\;}\times n_{0}}$. Statistical features are also calculated point-by-point [24]. The standard central moments of point (i,j) are defined as:

(2) $$M_{ij}^{I}=\frac{E\left[{\left(X_{ij}\left(t\right)-E\left[X_{ij}\left(t\right)\right]\right)}^{I}\right]}{\sigma^{I}},$$

where I refers to the order of statistical moments. σ is the standard deviation of the temperature variation waveform on the point (i,j). Skewness is the third standard central moment while kurtosis refers to the fourth moment. Therefore, it is simple to obtain skewness $Skew_{ij}$ and kurtosis $Kurt_{ij}$ for each point. Then, the skewness feature $Skew_{PT}\in R^{m\times n}$ and kurtosis feature $Kurt_{PT}\in R^{m\times n}$ are also reconstructed. The same analysis was also carried out on VT data to obtain $Skew_{VT}$ and $Kurt_{VT}$.

#### 3.2.2. Feature Selection Stage

In the feature selection stage, feature images of each modality are selected for the fusion step. A criterion based on the intensity contrast of the deformation area is implemented for feature selection. The feature images of PT could be denoted as $FI_{PT}\in \left\{X_{PT}\left(t\right),{\dot{X}}_{PT}\left(f\right),Skew_{PT},\;Kurt_{PT}\right\}$, then the intensity contrast of the deformation area is defined as:

(3) $$IC_{PT}=\frac{FI_{PT}^{De}-FI_{PT}^{No}}{FI_{PT}^{No}},$$

where $FI_{PT}^{De}$ refers to the average intensity of pixels on the deformation area and $FI_{PT}^{No}$ refers to the average intensity of pixels on the region without deformation. Thus, the selected feature image $F_{PT}\in R^{m\times n}$ of PT is expressed as:

(4) $$F_{PT}=\underset{FI_{PT}}{\max}IC_{PT},$$

The same procedure is also carried out on the VT data to obtain the feature image $F_{VT}\in R^{m_{0}\times n_{0}}$ for fusion.

#### 3.2.3. Feature Fusion Stage

In the feature fusion stage, data registration process is conducted to ensure that the feature images are in strict alignment. The size, edges and corners of samples are matched using rotating, scaling, and cropping operators. Then, the aligned feature images $F_{PT}\in R^{m\times n}$ and $F_{VT}\in R^{m\times n}$ are decomposed with multi-level NSST. Low-frequency sub-bands ($L_{PT}$,$\;L_{VT}$) and high frequency sub-bands ($H_{PT}^{l,d}$, $H_{VT}^{l,d}$) are generated as follows:

(5) $$L_{PT},H_{PT}^{l,d}=NSST\_decomp\left(F_{PT}\right),$$

(6) $$L_{VT},H_{VT}^{l,d}=NSST\_decomp\left(F_{VT}\right),$$

where l and d refer to the level and direction of high-frequency sub-bands. The intrinsic property values of low-frequency sub-bands are computed as:

(7) $$IP_{PT}=\mu_{PT}+Me_{PT},$$

(8) $$IP_{VT}=\mu_{VT}+Me_{VT},$$

where μ and Me represent the mean value and the median value of $L_{PT}$ and $L_{VT}$, respectively. The energy attribute functions are calculated by:

(9) $$E_{PT}\left(i,j\right)=e^{\alpha \left|L_{PT}\left(i,j\right)-IP_{PT}\right|},$$

(10) $$E_{VT}\left(i,j\right)=e^{\alpha \left|L_{VT}\left(i,j\right)-IP_{VT}\right|},$$

where α denotes the modulation parameter. Then, the fused low-frequency sub-band $L_{F}$ is obtained by a weighted mean:

(11) $$L_{F}\left(i,j\right)=\frac{E_{PT}\left(i,j\right)L_{PT}\left(i,j\right)+E_{VT}\left(i,j\right)L_{VT}\left(i,j\right)}{E_{PT}\left(i,j\right)+E_{VT}\left(i,j\right)}.$$

For the high-frequency sub-bands, the multi-scale morphological gradient of each sub-band needs to be calculated first. A multi-scale structuring element is defined as:

(12) $$SE_{t}=SE_{1}⊕SE_{1}⊕\ldots ⊕SE_{1},$$

where $SE_{1}$ refers to a basic structure element and t denotes the number of scales. ⊕ is the morphological dilation operator. The gradient of each high-frequency sub-band is calculated by:

(13) $$G_{PT,t}^{l,d}\left(i,j\right)=H_{PT}^{l,d}\left(i,j\right)⊕SE_{t}-H_{PT}^{l,d}\left(i,j\right)⊙SE_{t},$$

(14) $$G_{VT,t}^{l,d}\left(i,j\right)=H_{VT}^{l,d}\left(i,j\right)⊕SE_{t}-H_{VT}^{l,d}\left(i,j\right)⊙SE_{t}.$$

where ⊙ is the erosion operator. Thus, the linking strength is calculated by:

(15) $$\beta_{PT}^{l,d}\left(i,j\right)=\sum_{t=1}^{3}w_{t}G_{PT,t}^{l,d}\left(i,j\right).$$

(16) $$\beta_{VT}^{l,d}\left(i,j\right)=\sum_{t=1}^{3}w_{t}G_{VT,t}^{l,d}\left(i,j\right).$$

where $w_{t}=\frac{1}{2t+1}$ represents the weight of gradient in t-th scale. In this study, the scale of the morphological gradient is set as 3. PCNN is adapted for pixelwise fusion on the high-frequency sub-bands from PT and VT. PCNN generates parameters $T_{PT}^{l,k}\left(i,j\right)$ and $T_{VT}^{l,k}\left(i,j\right)$ for each pixel using the linking strength calculated by Equations (12)–(16) and high-frequency sub-bands obtained by Equations (5) and (6):

(17) $$T_{PT}^{l,d}\left(i,j\right)=PCNN\left(H_{PT}^{l,d}\left(i,j\right),\;\beta_{PT}^{l,d}\left(i,j\right)\right),$$

(18) $$T_{VT}^{l,d}\left(i,j\right)=PCNN\left(H_{VT}^{l,d}\left(i,j\right),\;\beta_{VT}^{l,d}\left(i,j\right)\right).$$

Then, the fused high-frequency sub-bands are obtained by:

(19) $$H_{F}^{l,d}\left(i,j\right)=\{\begin{array}{c} H_{PT}^{l,d}\left(i,j\right),\;\;\;\;\;\;\;if\;T_{PT}^{l,d}\left(i,j\right)\geq T_{VT}^{l,d}\left(i,j\right),\\ H_{VT}^{l,d}\left(i,j\right),\;\;\;\;\;\;\;\;\;\;\;\;\;\;\;\;\;\;\;\;\;\;\;\;\;\;\;\;\;\;otherwise. \end{array}$$

Finally, the fusion result can be reconstructed through the inverse NSST as follows:

(20) $$Fuse=NSST\_recon\left(L_{F},\;H_{F}^{l,d}\right).$$

## 4. Results and Discussion

### 4.1. PT Features and Quantitative Evaluation

**Figure 6:**
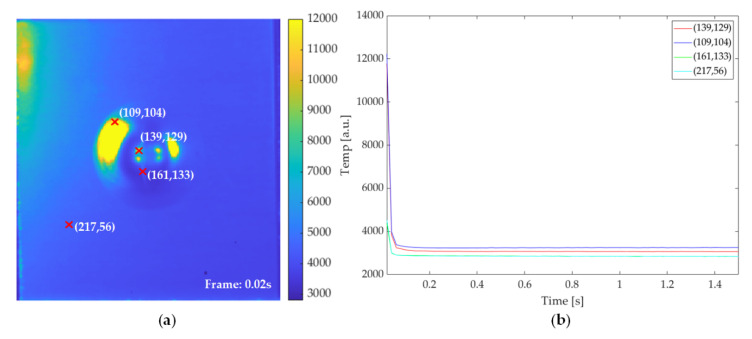
PT result on Al_G_60J sample: (**a**) pseudo color image of the first frame of the cooling stage; (**b**) temperature profile curve of the selected points.

Figure 6a shows the pseudo color image of the first frame of the cooling stage on the thermal sequence collected from the PT experiment on the Al_G_60J sample. Several typical information items could be observed in this frame. First, the impact-induced deformation includes the central area and the area surrounding the deformation. These two parts are divided by a ring-like low temperature area. Second, the huge impact energy caused a crack near the center of impact-induced deformation. The crack appears to be a slim and blurry strip crossing the central area of impact-induced deformation. Then, in order to investigate the transient property of these typical areas, we selected four points on these areas and illustrated the temperature curve in Figure 6b. It can be easily observed that, in the PT experiment, the central area and surrounding deformation show a higher temperature than the other parts. Moreover, the surrounding deformation has the highest temperature. The central area with high temperature refers to the debond between the front aluminium foil and the impregnated glass fibres. The surrounding deformation area represents the delamination between the rear aluminium layer and the impregnated glass fibres. Since the infrared sequence was collected from the back side of the testing sample, the around deformation area showed a higher temperature than the central area. It is worth noting that the temperature curve of the ring-like region is similar to the region without deformation. This is mainly because the ring-like area is the boundary of the impact center, while it still remains cemented without delamination and debonding. Using the temperature curve, we could simply find the time with the highest thermal contrast, and select the frame from the thermal sequence corresponding to this time point as the time-domain feature.

**Figure 7:**
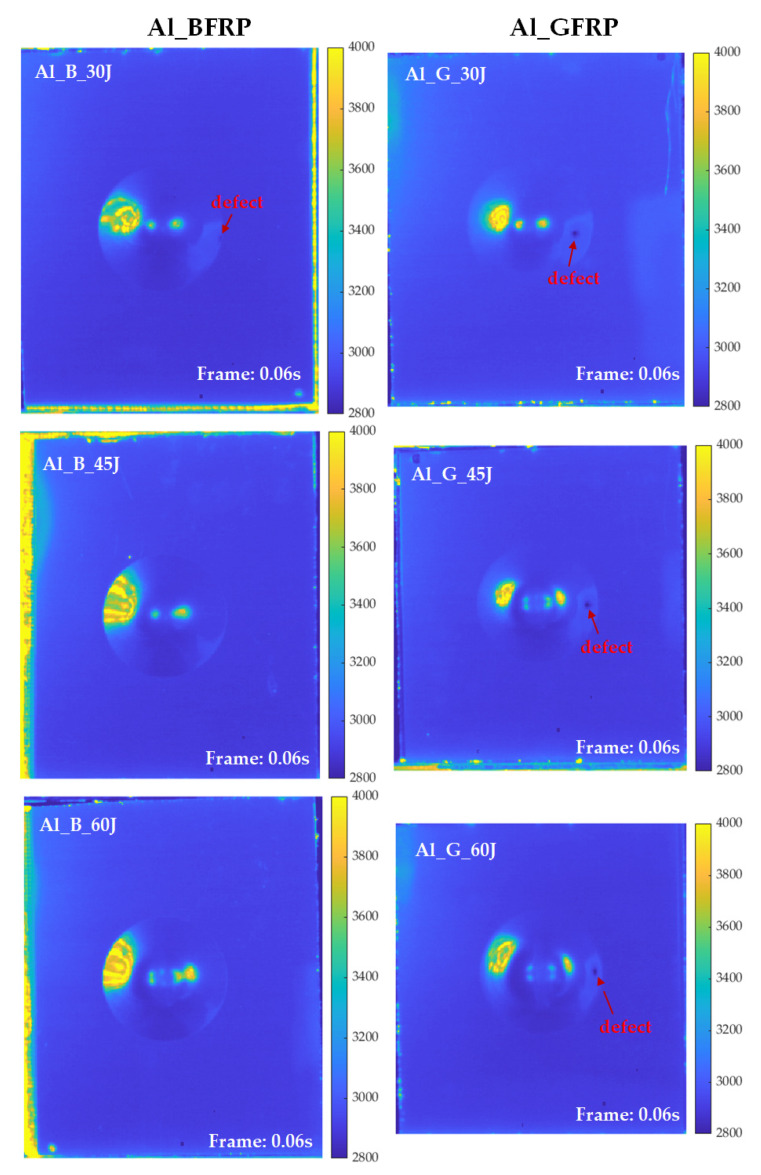
Typical frames from thermal sequences collected from PT experiments on six samples.

As illustrated in Figure 7, typical frames of six FMLs selected from PT experiments record the effective transient features of the cooling stage. It can be observed that the impact-induced deformation area as well as the central region does not change saliently with the increase of the impact energy. However, it is interesting that the ring-like firmly adhered region expands with the increasing impact energy. Moreover, the defects that appeared on the deformation area are detected on the time domain features. These defects are caused by fibre distortion during the low-velocity impact process. Another significant phenomenon is that no surface crack exists on the FMLs except for the Al_G_60J sample. This shows that the FMLs reinforced with glass fibres more easily attain the energy level causing the first crack.

**Figure 8:**
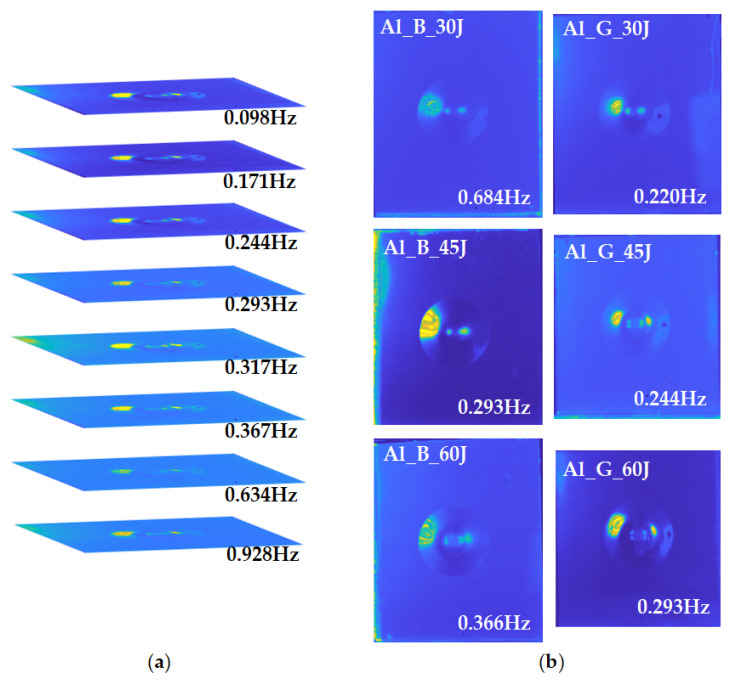
Frequency-domain analysis of the PT experiments on the six samples: (**a**) amplitude imaging on different frequency points of Al_G_60J sample; (**b**) typical amplitude imaging results on six samples.

In order to extract sufficient features from the original thermal sequence, the authors conduct frequency-domain analysis and statistical analysis on the thermal sequence. Figure 8a records the amplitude imaging results on the Al_G_60J sample. The amplitude images obtained from low-frequency correspond to the overall defect contour information, while the high-frequency band preserves the detailed information from the surface layer. Amplitude images calculated from the PT experiments on six FMLs are illustrated in Figure 8b. It is obvious that features from the frequency domain have a smoother temperature distribution, which avoids the high temperature blurry region caused by heat accumulation.

**Figure 9:**
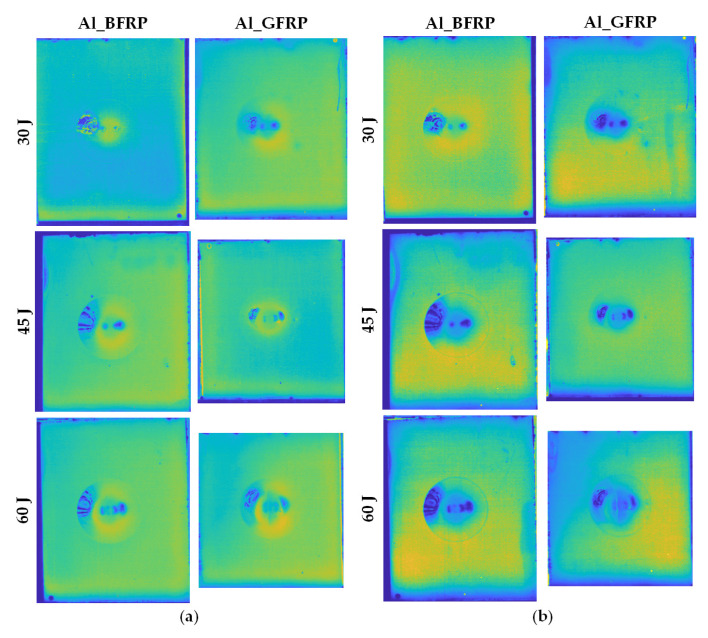
Statistical features of the PT experiments on six samples: (**a**) skewness features; (**b**) kurtosis features.

The statistical features of these FMLs are illustrated in Figure 9. The statistical features contribute significantly to the contrast improvement on the anomaly area of the PT data. Skewness is sensitive to the symmetry of the temperature variation curve, while kurtosis measures the relative flatness of the temperature variation curve in relation to the shape of a normal distribution. As shown in Figure 9, kurtosis features extracted from the PT experiments provide more defect information than skewness features; this is true above all for the boundary of the deformation area. It is worth noting that the boundary of the deformation area detected by kurtosis features on the basalt fibre reinforced FMLs has a higher contrast than the glass fibre reinforced FMLs. Besides, the boundary of the deformation area detected by the skewness features provides auxiliary information on the Al_B_30J sample.

**Figure 10:**
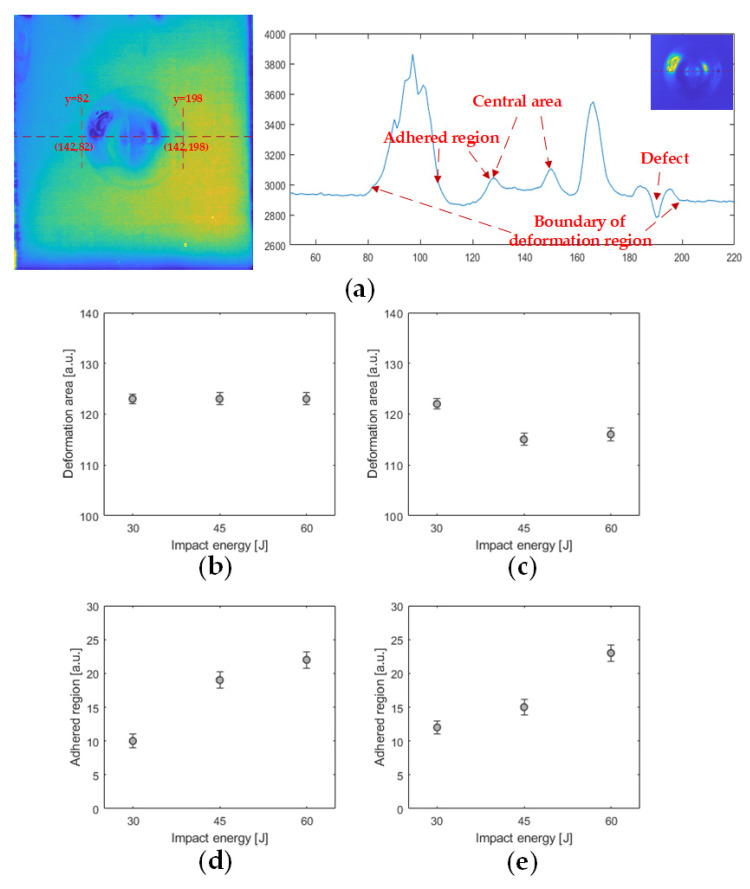
Quantitative analysis of the PT experiments: (**a**) analysis on the temperature curve of the Al_G_60J sample; (**b**) deformation area of Al_B samples; (**c**) deformation area of Al_G samples; (**d**) concave area of Al_B samples; (**e**) concave area of Al_G samples.

For a more accurate assessment on the impact-induced deformation area, a quantitative analysis is carried out. As illustrated in Figure 10a, the kurtosis feature provides distinct boundary information of the deformation area. This could be utilized for a quantitative analysis on the deformation size. First, since the low-velocity impact induced deformation is circular, two parallel vertical lines are drawn tangent to the deformation edge at points (142,82) and (142,198). Then, the horizontal line x=142 is adapted to generate the temperature distribution curve. We made the horizontal line at the same location of the time-domain feature and analyzed the temperature distribution. Finally, comparing both the thermal contrast and the temperature distribution, the range of the central deformation area, a ring-like firmly adhered region and the surrounding deformation area could be specified. The size of the deformation could be quantitatively analyzed by calculating the distance of coordinates corresponding to the temperature distribution curve. As shown in Figure 10b,c, the width of the deformation area does not change significantly with the variation of the impact energy and fibre reinforced materials. However, Figure 10d,e shows that, with the increase of impact energy, the area of the ring-like firmly adhered region around the central deformation on both basalt and glass fibre reinforced FMLs expands obviously. The expansion of the width of these regions without debonds around the central deformation is positively correlated to the impact energy.

### 4.2. VT Features

**Figure 11:**
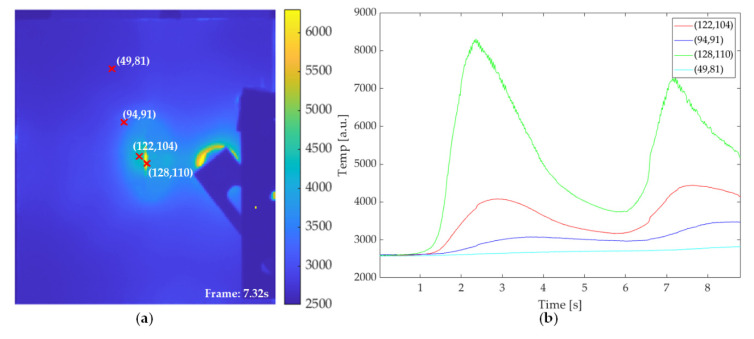
VT result on Al_G_60J sample: (**a**) pseudo color image of the first frame after two cycles of excitation; (**b**) temperature profile curve of the selected points.

Figure 11a illustrates the first frame after two periods of lock-in ultrasonic wave excitation on the VT experiment on the Al_G_60J sample. Compared with the PT experiment, the frame extracted from the VT experiment data preserves less information on the deformation area, while the higher contrast is achieved on the crack which exists on the rear aluminium layer. As shown in Figure 11b, the temperature variation curve on the selected point from the crack is significantly different from the other location. The heat concentration on the cracks leads to an extremely high contrast on this type of defect. Thus, cracks can easily be recognized by the VT experiment. The average temperature of points selected from the deformation area is higher than other parts. This clue underlines the existence of debond on the deformation area.

**Figure 12:**
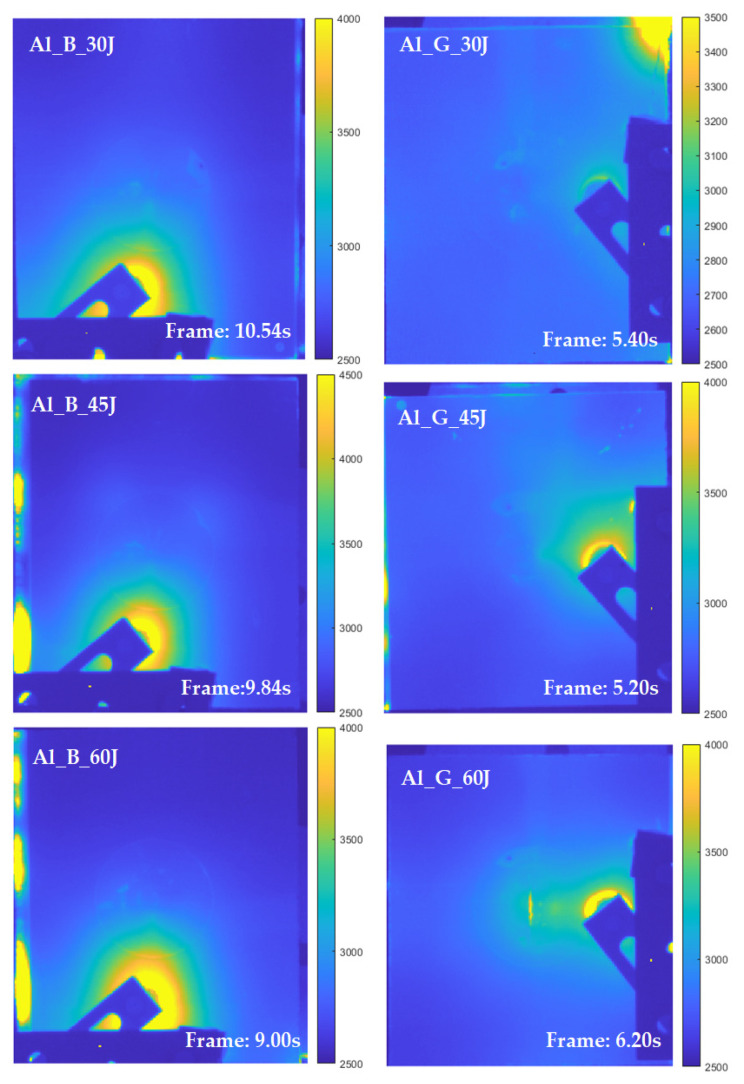
Typical frames from thermal sequences collected from VT experiments on six samples.

As illustrated in Figure 12, typical frames of six FMLs are obtained on the cooling stage after the first and second period of the ultrasonic wave excitation. It can be observed that, on the low-velocity impact deformation area, the central deformation area is not as clear as on the PT experiment. However, the high contrast crack information and some defects could be an essential complement to the results of the PT experiment. Moreover, with the increasing impact energy, the central deformation area and the ring-like adhered region become clear (e.g., the Al_B_60J and Al_G_45J in Figure 12).

**Figure 13:**
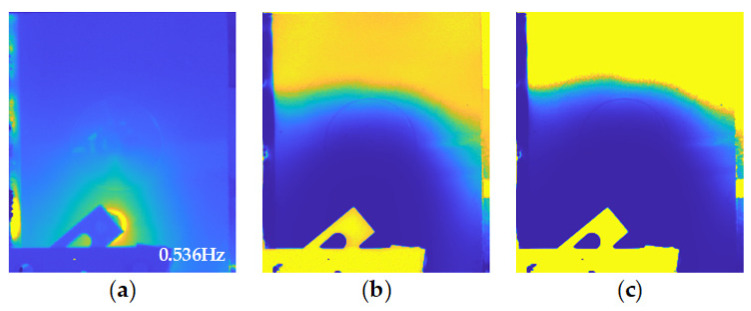
Features extracted from the VT experiment on Al_B_60J sample: (**a**) amplitude imaging feature; (**b**) skewness feature; (**c**) kurtosis feature.

As in the PT experiment, the feature extraction process using frequency analysis and statistical analysis is also conducted to obtain sufficient features. As an example, features on the Al_B_60J sample are illustrated in Figure 13. It is obvious that the frequency-domain feature (Figure 13a) effectively suppresses the high temperature blurry region which has a negative influence on the thermal contrast of the impact-induced deformation area. However, statistical features (Figure 13b,c) do not show a promising performance on the defect information extraction and enhancement. Skewness and kurtosis are statistics with high sensitivity to the difference between the temperature variation curves obtained from pixels. Considering the excitation characteristics of vibro-thermography, the area near the transducer is heated earlier than other parts of this sample. This result shows clearly the non-uniform heating process induced to the sample. Therefore, with the process of thermal transmission, the statistical features calculated on the temperature variation curve of the pixel would change with the distance from the excitation area.

### 4.3. Feature Fusion Imaging

Feature selection based on the intensity contrast of the deformation area is conducted, and the selected features of each excitation modality are illustrated in Table 3.

**Figure 14:**
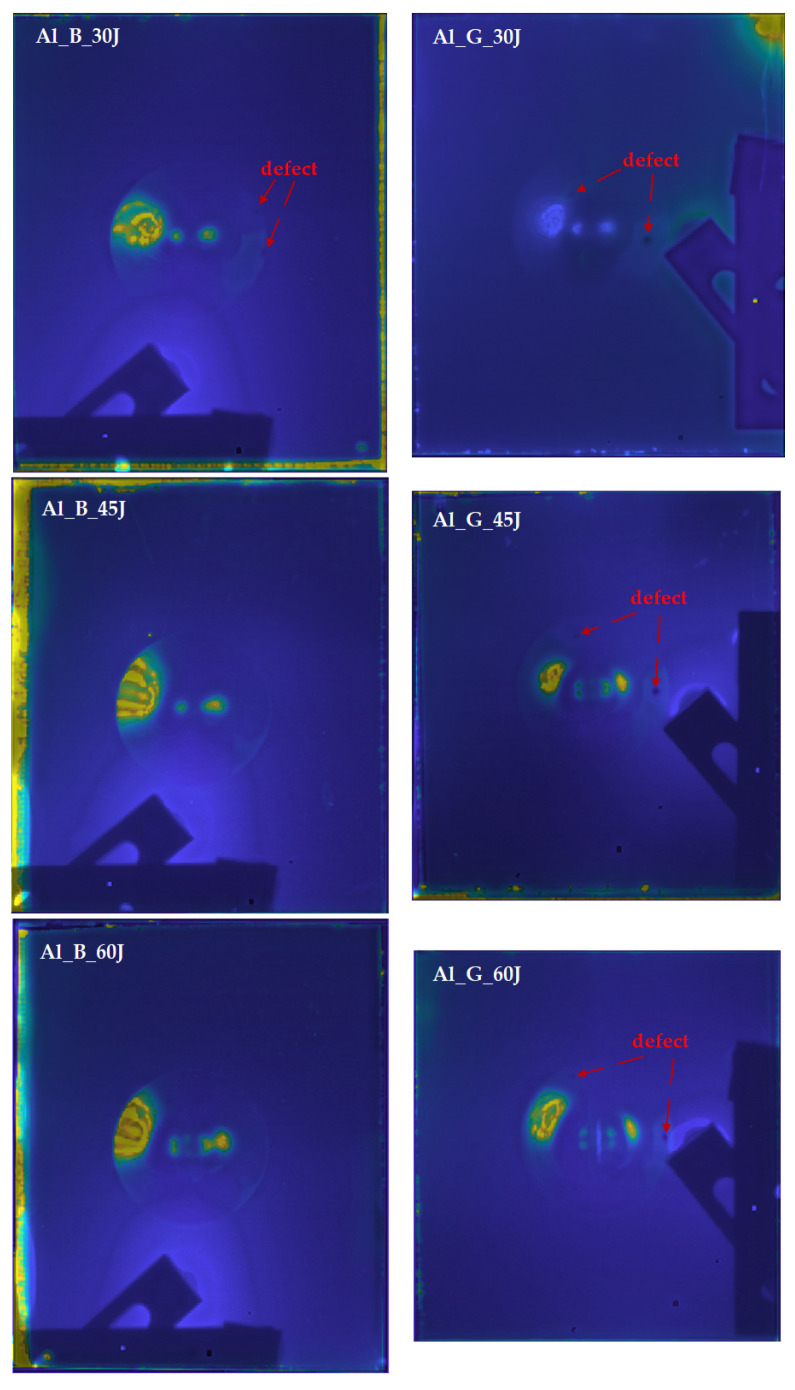
Fusion results of the proposed FE-S-F framework on six FMLs samples.

The selected feature images are fused with the NSST-PCNN method, and the final outputs of the proposed FE-S-F framework are shown in Figure 14. Several interesting enhancements can be observed on the fusion results. First, the fusion results contain the defect information from two excitation modalities. For example, the defects marked in Figure 14 combine the defect information detected by both the PT and VT. The information of the fibre distortion which occurred at different depths on the rear side of the aluminium panel is collected by these two modalities and then fused with the proposed FE-S-F framework. Second, on the Al_G_60J sample, not only more defects are detected when compared with only the PT experiment, but also the contrast on the crack is saliently improved. Regarding the result of PT experiment, the crack is a slim and blurry strip, while on the fusion result, the morphology of the crack can be observed clearly. The latter result is essential for crack inspection and evaluation of FMLs. Third, the boundary of the deformation area on six FMLs is clearer than what was observed with only the single infrared excitation experiment. Since the fusion results contain the information from two excitation modalities, this effectively enhances the information on the deformation area. Besides, the high temperature blurry regions, such as the surrounding deformation area (debond between rear aluminium layer and fibre reinforced materials) on the PT and area near the excitation transducer on VT, are suppressed on the fusion results to provide better thermal contrast on the central deformation area.

## 5. Conclusions

In this paper, a multi-excitation infrared fusion method was proposed for the non-destructive evaluation of FMLs. OET with a superior performance on revealing surface and subsurface defects was combined with VT to improve the ability of defect detection. For the purpose of utilizing the information of two excitation modalities, a fusion framework FE-S-F containing feature extraction, feature selection, and fusion was introduced for defect information extracting and merging. Experiments using IRT with two excitation modalities were carried out on six FMLs including aluminium-BFRP/GFRP. Raw data collected from experiments were further investigated with the fusion framework FE-S-F. Features on the time-domain, frequency-domain, and statistic equational perspective were extracted from the raw data. Statistical features of PT data showed a high contrast on the boundary of the deformation area and were adapted for the quantitative analysis of the deformation area. Temperature distribution curves of PT data were investigated for assessment on the size of the deformation area and the ring-like adhered area. We found that the width of the deformation area did not change significantly with the variation of the impact energy and fibre reinforced materials, while the expansion of the adhered region was directly proportional to the impact energy. Moreover, the FMLs reinforced with glass fibres were found to more easily attain the energy level causing the first crack. The fusion results on the feature images selected by intensity contrast on the deformation area effectively combined the defects from different excitations. The information provided by the VT experiment is complementary to the PT experiment on fusion results, and the final fused images have a higher contrast on the crack as well as the deformation area than what was observed with only the single infrared excitation experiment. The feature selection depends on the intensity contrast of deformation area, which limits the application of the proposed method on blind scanning inspection when the deformation is not obvious but internal defects exist. An effective unsupervised feature selection method will be the focus of follow-up work.

## Figures and Tables

**Table 1:** Summary of the mechanical properties of raw materials.

Materials	Modulus of Elasticity (GPa)	Ultimate Tensile Strength (MPa)	Elongation at Break (%)
Aluminium 2024-T3	73.1	483.0	18
Basalt fibres	89	3530	3.15
Glass fibres	77	3450	4.7

**Table 2:** FMLs samples manufactured.

Remark of the Laminates	Stacking Sequence	Thickness [mm]	Areal Density [kg/m^3^]	Fibre Volume Fraction	Metal Volume Fraction
Al_B	$Al/B_{10}/Al$	3.03±0.04	6.31±0.10	0.45±0.01	0.39±0.01
Al_G	$Al/G_{10}/Al$	2.89±0.04	6.22±0.05	0.48±0.01	0.41±0.01

**Table 3:** Feature images after feature selection on two modalities.

FMLs	Features from PT	Features from VT
Al_B_30J	Time	Frequency
Al_B_45J	Frequency	Frequency
Al_B_60J	Time	Frequency
Al_G_30J	Frequency	Frequency
Al_G_45J	Time	Frequency
Al_G_60J	Time	Frequency

## Data Availability

Data sharing not applicable.
